# Revisiting the LSER Approach in the Era of Machine Learning: Insights from IAM Chromatography

**DOI:** 10.1021/acs.molpharmaceut.6c00381

**Published:** 2026-08-05

**Authors:** Wiktor Nisterenko, Katarzyna Ewa Greber, Magdalena Kierkowicz, Krzesimir Ciura

**Affiliations:** 1 Department of Physical Chemistry, Faculty of Pharmacy, 37804 Medical University of Gdansk, Al. Gen. J. Hallera 107, 80-416 Gdansk, Poland; 2 636870 UCB, 216 Bath Road, Slough SL1 3WE, United Kingdom

**Keywords:** QSRR, immobilized artificial membrane, machine learning, linear solvation energy relationship, ionization descriptors

## Abstract

The present study demonstrates the integration of the linear solvation energy relationship (LSER) concept with machine learning (ML) methodologies to improve the predictive and interpretative capabilities of chromatographic retention modeling. Immobilized artificial membrane (IAM) chromatography was employed as a model biochromatographic system, and an in-house library of 993 structurally diverse compounds, with experimentally determined chromatographic hydrophobicity index of IAM (CHI_IAM_), was used to train LSER-ML models. LSER descriptors were calculated using Absolv and extended with ionization-state descriptors to evaluate the applicability of the LSER framework for realistic in silico virtual screening scenarios. Several regression algorithms were tested, including linear, neighborhood-based, kernel-based, and ensemble tree-based models. Among them, the support vector regression with the radial basis function kernel (SVR-RBF) demonstrated the most balanced performance across all validation metrics of *R* ^2^_train_ = 0.884, *R* ^2^_test_ = 0.853, and *Q* ^2^_cv_ = 0.811, achieving predictive errors (RMSE_train_ = 5.546, RMSE_test_ = 4.609, and RMSE_cv_ = 6.989) close to the analytical uncertainty. Model interpretability was achieved using SHapley Additive exPlanations (SHAP), which confirmed the mechanistic relevance of the Abraham descriptors and the dominant contribution of hydrophobic volume and hydrogen-bonding properties to IAM retention. Applicability domain was verified with a Williams plot (±3 standardized residuals and leverage threshold h*). The results indicate that the proposed LSER-ML approach provides an interpretable, robust, and generalizable tool for modeling membrane-mimetic chromatographic systems and can be effectively applied in virtual screening and property-based molecular design.

## Graphical abstract

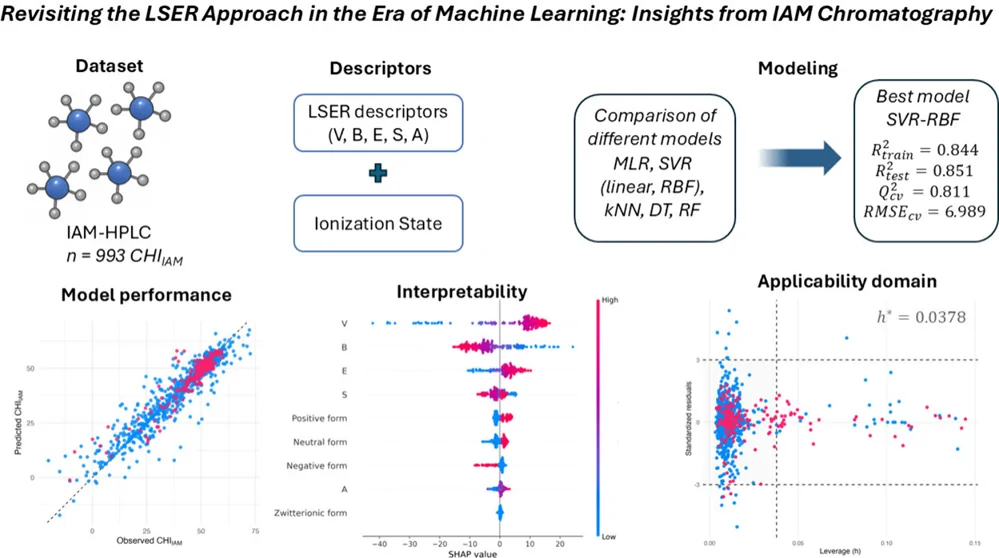

## 1 Introduction

The relationship between molecular structure and chromatographic retention has long fascinated analytical chemists, as it provides a rational basis for understanding separation mechanisms and predicting retention behavior. Establishing reliable structure–retention relationships enables more efficient optimization of separation conditions, reduces the number of experimental trials required, and supports the identification of unknown analytes.^1^ The capability to predict retention from molecular descriptors has therefore been central to the development of quantitative structure–retention relationship (QSRR) models, which bridge molecular-level information and chromatographic observables.

Among the conceptual frameworks that have shaped QSRR development, the linear solvation energy relationship (LSER), formulated by Abraham and co-workers,^2,3^ has played a particularly influential role. In its general form for transfer processes between two condensed phases, the solvation parameter model is expressed as

1 log⁡SP=c+eE+sS+aA+bB+vV

where SP is a free energy-related property, such as a retention factor or partition constant, and the capital letters denote solute descriptors: *E* represents the excess molar refraction, capturing polarizability arising from n- and π-electron interactions; *S* quantifies dipolarity/polarizability, reflecting orientation and induction interactions; *A* and *B* represent overall hydrogen-bond acidity (donor capacity) and basicity (acceptor capacity), respectively; and *V* is McGowan’s characteristic volume, which accounts for cavity formation and residual dispersion interactions upon solute transfer between phases. The lower-case italic letters (*e*, *s*, *a*, *b*, *v*) are system constants that encode the complementary properties of the biphasic system involved in the transfer process. In chromatographic applications, they reflect the difference in interaction properties between the mobile and stationary phases and are independent of the solute.^3^ The model intercept, *c*, absorbs contributions from the phase ratio and any systematic offset. LSER not only provides predictive models of retention but also offers mechanistic insight into solvation and partitioning phenomena occurring during chromatographic separation and has therefore been widely used for chromatographic system and column comparison.^4–16^

Importantly, LSER models have been successfully extended beyond purely chromatographic applications.^13,17–19^ Because the physicochemical descriptors involved also govern solute transfer processes in biological environments,^19^ LSER provides a valuable insight into biological partitioning phenomena.^20^ Hence, the approach can serve as a unifying framework for comparing physicochemical-based approaches like chromatographic and biological processes from the same physicochemical perspective.

Among biomimetic chromatographic systems, immobilized artificial membrane (IAM) chromatography occupies a particularly prominent position in pharmaceutical research and drug discovery.^21–23^ The IAM stationary phase, consisting of phosphatidylcholine moieties covalently immobilized on a silica support, mimics the physicochemical environment of biological membranes and provides a reproducible, high-throughput surrogate for membrane partitioning. The IAM-derived affinity to phospholipids has been shown to correlate with a broad range of pharmacokinetic end points, including steady-state volume of distribution,^24^ as well as cardiotoxicity risk assessed via hERG channel affinity.^25^ Most notably, IAM retention parameters correlate with passive membrane permeability as determined by PAMPA assays, providing a cell-free alternative for early permeability screening.^26,27^ This breadth of pharmacologically relevant correlations, combined with the experimental simplicity and high throughput of the fast gradient IAM-HPLC protocol introduced by Valkó and co-workers,^28^ makes IAM chromatography a cost-effective and widely adopted tool in early drug discovery pipelines, in both academia and industry.^29,30^

In the current era of data-driven modeling, the growing availability of large, high-quality chromatographic data sets opens new opportunities for revisiting the LSER concept using machine learning (ML) tools. A key enabler of this opportunity is the availability of computational tools for calculating Abraham model solute descriptors (*E*, *S*, *A*, *B*, *V*) directly from molecular structure. Cheminformatics modules such as Absolv (ACD/Labs Percepta) or AbraLlama^31^ models allow for their calculation from molecular structure, opening LSER-based modeling to novel chemical entities for which no experimental descriptor data exist, a critical requirement for virtual screening in drug discovery. Atapattu and co-workers recently confirmed that computationally derived Abraham descriptors can be successfully employed within the solvation parameter model across various chromatographic systems, supporting their practical use in predictive modeling.^27,32^ The second aspect concerns model development: traditional multiple linear regression (MLR), though historically dominant, may be insufficient for capturing nonlinear structure–retention relationships in structurally diverse data sets. These limitations can be effectively addressed through modern ML algorithms, which offer greater flexibility while preserving the mechanistic interpretability inherent to the LSER framework.

The present study aims to demonstrate that the LSER paradigm can be effectively integrated with modern ML methodologies to improve both predictive accuracy and mechanistic interpretability. Using IAM chromatography as a model biochromatographic system, we constructed and compared a series of ML-based LSER models trained on a library comprising 993 structurally diverse compounds. LSER descriptors were calculated and augmented with ionization-state descriptors, following the approach of Rosés and co-workers,^33^ to extend the framework to ionizable drug-like compounds and evaluate its applicability in virtual screening workflows. The tested algorithms represented diverse regression paradigms, including linear, neighborhood-based, kernel-based, and tree-based ensemble models. Finally, the best-performing model was subjected to rigorous validation following the Organization for Economic Co-operation and Development (OECD) principles,^34^ ensuring robustness, reproducibility, and domain applicability, as well as mechanistic interpretation using SHapley Additive exPlanations (SHAP).^35^

## 2 Materials and Methods

### 2.1 Data Set

This study uses a unified data matrix built from IAM-HPLC measurements of the chromatographic hydrophobicity index (CHI_IAM_). The matrix integrates two sources: (i) a series of sulfonamide derivatives reported by Nisterenko et al.^36^ and (ii) previously published data for the small molecules, reported as the reference set from Ciura, 2024.^37^ In total, the data set comprises 993 compounds. The complete descriptor matrix, including compound names and corresponding SMILES, is provided in Table S1 in the Supporting Information.

### 2.2 LSER and Ionization Descriptors

LSER descriptors were calculated by using the Absolv module of ACD/Labs Percepta (version 2024.1.3). In addition, the PhysChem module of the same software package was employed to estimate the ionization states of the target analytes under experimental conditions. All calculated descriptors are summarized in Table S2 in the Supporting Information.

### 2.3 Principal Component Analysis (PCA)

Principal component analysis (PCA) was applied to assess the data structure and the chemical space distribution. PCA was computed on the LSER descriptors previously centered and scaled to unit variance. Two complementary visualizations were produced: (i) a standard score plot (PC1 vs PC2) to display sample distribution in the chemical space, and (ii) a biplot overlaying scores with variable loadings (arrows) to indicate the primary descriptor directions. PCA was performed with R 4.4.3.

### 2.4 Model Development

We compared a diverse panel of supervised regression algorithms that included multiple linear regression (MLR), support vector regression (SVR; linear and radial-basis kernels), k-nearest neighbors (kNN), a single decision tree (DT), random forest (RF), and LightGBM. All models were implemented as reproducible pipelines that couple preprocessing, hyperparameter tuning, and evaluation.

#### 2.4.1 Preprocessing

The data set was divided into a training set (80%) and an external test set (20%) using the Kennard–Stone algorithm in descriptor space to ensure uniform coverage of the chemical domain. Numeric descriptors served as inputs. For algorithms sensitive to the feature scale (MLR, SVR, kNN), features were *z*-standardized after imputation. Tree-based models (DT, RF, and LightGBM) were trained on unscaled descriptors. The response (CHI_IAM_) was kept on its original scale.

#### 2.4.2 Performance Metrics

Each model was evaluated using the coefficient of determination (*R* ^2^) and root mean squared error (RMSE) on the training, test set, and cross-validated *R* ^2^, denoted as *Q* ^2^_cv_ (mean of the *Q* ^2^_(k)_ from each *k*-fold). These metrics are defined as follows:

2 R2=1−∑i=1n(yi−yi^)2∑i=1n(yi−y̅)2

3 $$Q_{\left(k\right)}^{2}=1-\frac{\underset{i\in V_{k}}{\sum }{\left(y_{i}-{ŷ}_{i,\left(k\right)}\right)}^{2}}{\underset{i\in V_{k}}{\sum }{\left(y_{i}-{\mathrm{y̅}}_{\mathrm{train}}\right)}^{2}}$$

4 $$Q_{\mathrm{cv}}^{2}=\frac{1}{K}\overset{K}{\underset{k=1}{\sum }}Q_{\left(k\right)}^{2}$$

5 $$Q_{\mathrm{SD}}^{2}=\sqrt{\frac{1}{K-1}\overset{K}{\underset{k=1}{\sum }}{\left(Q_{\left(k\right)}^{2}-Q_{\mathrm{mean}}^{2}\right)}^{2}}$$

6 RMSE=∑i=1n(yi−yi^)2n

where *n* represents the total number of samples, *y*_*i*_ is the experimental value of sample *i*, yi^ is the predicted value, and *y̅* is the mean value. *K* is the number of folds in *k*-fold cross-validation. *V*_*k*_ is the validation subset (fold) at iteration *k*. *ŷ*_*i*__,(*k*)_ is the out-of-fold prediction for sample *i* obtained from the model trained without *V*_*k*_ *.*

#### 2.4.3 Model Selection Framework

We used 5-fold cross-validation on the training split with grid search (scikit-learn GridSearchCV) to optimize hyperparameters. Cross-validation folds were assigned by random partitioning of the training split (scikit-learn KFold, shuffle = True, with a fixed random seed) and were not stratified with respect to sub-dataset membership, i.e., the sulfonamide series versus the reference small-molecule set. Because compounds from both series were therefore distributed across folds, the reported *Q* ^2^_cv_ may be somewhat optimistic relative to a scenario in which the model is applied to an entirely new chemical class; this concern is partially mitigated by the Kennard–Stone external test set, which provides an independent estimate of generalization across descriptor space. Unlike a random hold-out, the Kennard–Stone algorithm selects test compounds by iteratively maximizing their pairwise distance in descriptor space, so the resulting test set spans the boundaries and extremes of the chemical domain rather than merely resampling its densest regions. The hyperparameter grids explored for each algorithm were as follows: MLR (fit_intercept: [True, False]; positive: [False, True]), SVR with linear kernel (C: [0.1, 1, 10]; ε: [0.01, 0.1, 0.5]), SVR-RBF (C: [1, 10, 100]; γ: [“scale”, 0.01]; ε: [0.01, 0.1]), kNN (n_neighbors: [5–41, including values scaled to √*n* and 5–10% of training set size]; weights: [“uniform”]; distance metric p: [1, 2]; PCA variance retained: [None, 0.85, 0.90, 0.95]), DT (max_depth: [None, 8, 16]; min_samples_split: [2, 5, 10]; min_samples_leaf: [1, 2, 4]; max_features: [None, “sqrt”, 0.6]), RF (n_estimators: [300, 600]; max_depth: [None, 8, 16]; min_samples_split: [2, 5]; min_samples_leaf: [1, 2]; max_features: [“sqrt”, 0.6]), and LightGBM (n_estimators: [800, 1600]; num_leaves: [8, 16]; max_depth: [4, 6]; min_child_samples: [40, 100]; subsample: [0.7]; colsample_bytree: [0.7, 1.0]; reg_alpha: [0.0, 0.5]; reg_lambda: [1.0, 5.0]; learning_rate fixed at 0.03). Final selection of the best model was based on a joint criterion of high cross-validated *R* ^2^ (*Q* ^2^_cv_) together with strong external test *R* ^2^ while guarding against overfitting by inspecting the agreement among training, cross-validation, and test *R* ^2^ values. The complete modeling code, including all preprocessing pipelines, hyperparameter optimization, and the final trained models, is publicly available at https://github.com/BioChromML/LSER-ML.

#### 2.4.4 Interpretability and the Model’s Applicability Domain

For the best-performing model, we quantified feature contributions using permutation importance and SHAP to obtain complementary global rankings and local attributions (permutation importance plot provided in the Supporting Information, Figure S1).

The model’s applicability domain was assessed using a Williams plot, which relates standardized residuals to leverage values. Compounds were classified as outliers if their standardized residuals exceeded ±3 or if their leverage exceeded the critical threshold h* = 3­(*p* + 1)/*n*, where *p* denotes the number of descriptors and *n* the number of training set compounds.

All modeling was performed in Python 3.11.2, whereas data visualization and statistical analysis were carried out using R, the ggplot2 package in R 4.4.3 (free software).

## 3 Results and Discussion

The IAM stationary phase was selected as a model system due to its well-established ability to mimic physiological membrane partitioning.^21–23^ Unlike purely hydrophobic phases, the surface of the IAM stationary phase is covered by phosphatidylcholine moieties covalently bound to a silica support, which, under physiological pH, exhibits a zwitterionic character, thereby reproducing both polar and nonpolar interaction sites. This dual nature allows IAM retention to reflect a balance of solvation phenomena relevant to both chromatographic and biological systems, making it an ideal platform for the present LSER–ML study.

Additionally, IAM chromatography is highly reproducible, as confirmed by both inter- and intralaboratory studies, and the fast gradient protocols proposed by Valkó and co-workers^38^ allow for rapid and standardized determination of chromatographic hydrophobicity indices (CHI_IAM_).^28^ This combination of reproducibility, procedural robustness, and efficiency makes IAM chromatography an ideal source of high-quality experimental data suitable for ML applications. An additional advantage is that LSER models for IAM chromatography have already been successfully developed, including those based on gradient elution data obtained using the Valkó protocol.^38^

Building on this foundation, the present study integrates two IAM chromatographic data sets, one focused on a diverse set of small molecules representing various pharmacological and toxicological chemical classes^37^ and the other on a large series of anticancer sulfonamides.^36^ Both data sets were obtained under identical experimental conditions following the fast gradient IAM-HPLC protocol.^38^ As discussed in detail in our previous work,^37^ the first data set set incorporates CHI_IAM_ values predominantly generated in our laboratory, supplemented with literature data measured on both the original IAM.PC.DD2 columns (type A silica) and the newer batches (type B silica, introduced in 2018).

Valkó et al.^28^ demonstrated that CHI_IAM_ values from the two column generations are generally comparable, with differences limited to a small subset of polar basic compounds. Regarding the mobile phase composition, ammonium acetate was used in the majority of studies, while phosphate buffer at pH 7.4 was employed in two cases. Notably, the compounds investigated in those two studies (isoxazole^39^ and oleanolic acid derivatives^40^) exist predominantly in their neutral forms under the applied conditions, and the same calibration procedure with identical reference standards was applied across all studies, minimizing the potential impact of buffer-related differences on their retention. While minor deviations arising from differences in buffer type and column generation cannot be entirely excluded, particularly for ionizable compounds, the decision was made to retain all data points to maximize the size and chemical diversity of the training set, which is critical for the effective application of ML algorithms. In contrast, the sulfonamide data set^36^ was generated entirely in our laboratory on a single IAM.PC.DD2 column with ammonium acetate buffer, and is therefore free from the above-mentioned sources of variability.

After merging, the combined data set comprised 993 individual observations, representing the largest and most chemically diverse IAM retention libraries reported to date. The size and internal consistency of this data set enable the effective use of ML algorithms that rely on large, information-rich data matrices, thereby improving model generalization and predictive confidence. PCA performed on the combined data set confirmed that the chemical domains represented by the two experimental series are largely complementary (Figure 1A). Furthermore, linear trends visible on the PCA biplot (Figure 1B) indicated that Abraham descriptors can be quantitatively linked to the experimentally determined CHI_IAM_ values, supporting their suitability for LSER-based modeling.

**Figure 1:**
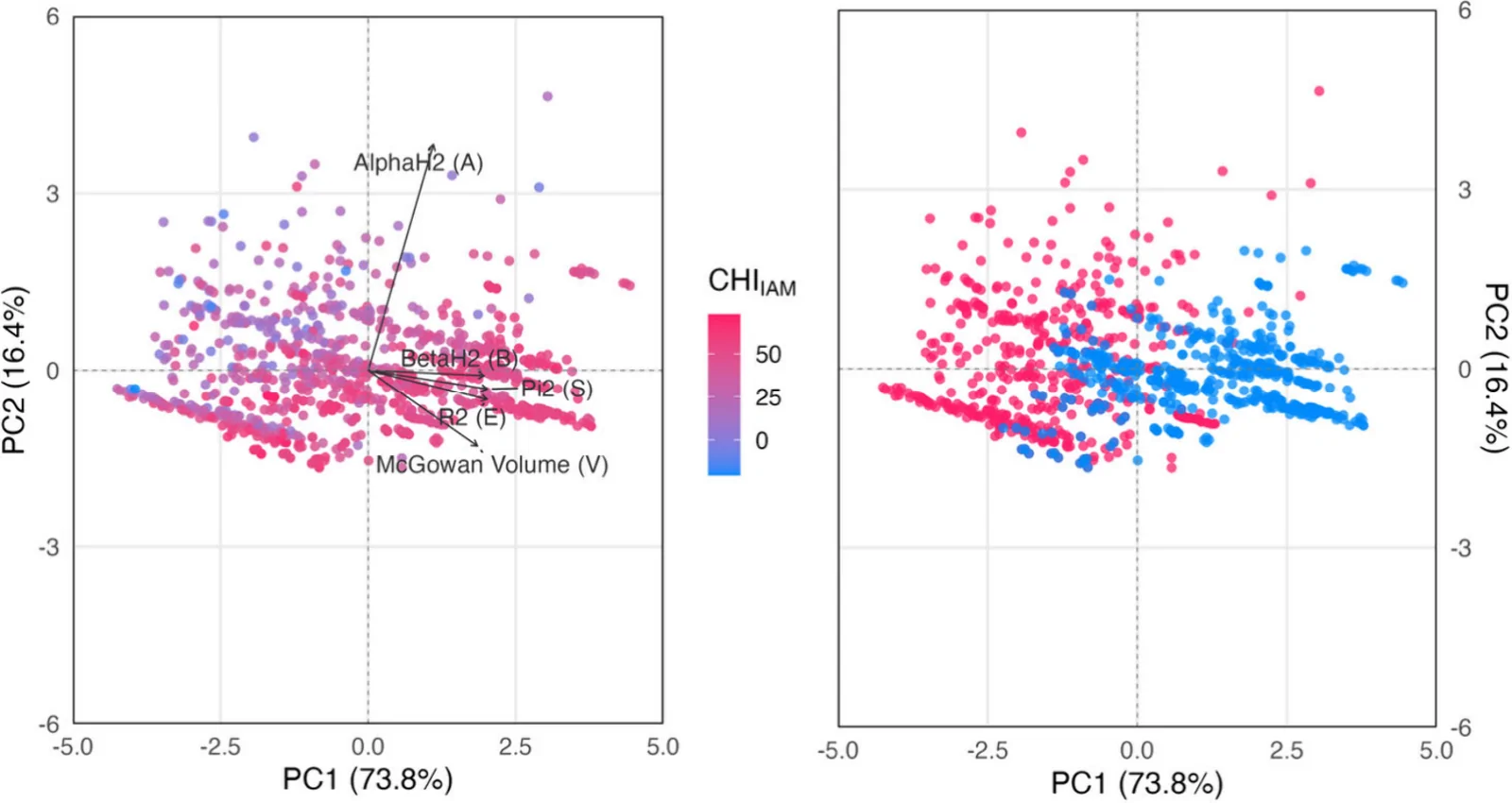
Principal component analysis (PCA) of the IAM data set based on Abraham solvation descriptors. (A, left) Biplot showing descriptor loadings (arrows) and sample distribution colored by experimental CHI_IAM_ values. (B, right) Score plot colored by data set origin (sulfonamides - pink vs reference set - blue), confirming complementary chemical space coverage.

The application of the solvation parameter model relies on certain assumptions, notably the additivity of individual interaction terms and the availability of high-quality descriptor values. As comprehensively reviewed by Poole,^41^ descriptor quality is a significant concern, since experimental descriptors determined from different sources and methods may vary considerably, and this dispersion directly affects model precision and the reliability of system constants. To address this issue in the present study, all descriptors were computed rather than sourced from experimental databases, ensuring consistency across the entire compound library, including newly synthesized molecules for which no experimental characterization exists. It should be stressed that Atapattu and co-workers recently confirmed the practical validity of computationally derived Abraham descriptors within the solvation parameter model.^27,32^ Nevertheless, the validation sets used in those studies were relatively small (approximately 40 compounds), all of which possessed well-characterized experimental descriptors available in established databases. This raises a methodological concern: computational descriptor tools are themselves calibrated on experimental databases, and evaluating their use on compounds already represented in those databases may introduce a form of circular reasoning, potentially inflating the apparent model performance. The present study addresses this limitation by operating at a substantially larger scale, nearly 1000 compounds, of which approximately half are newly synthesized drug candidates without any prior characterization in terms of Abraham descriptors, thereby reflecting a genuinely realistic virtual screening scenario in which computational descriptors are the only viable source of input data.

An additional consideration concerns the treatment of ionizable compounds. The classical LSER model was originally developed for neutral molecules,^10,12^ and the Absolv module employed in this study computes Abraham descriptors exclusively for the neutral form. However, the group of Rosés demonstrated that incorporating ionization information significantly improves model performance across multiple chromatographic systems, including reversed-phase (RP-LC),^42^ micellar (MLC),^11,18,43^ and microemulsion liquid chromatography (MELC).^17,35,46^ Since the majority of drug-like compounds are ionizable acids or bases that exist as mixtures of neutral and charged species under physiological conditions, the baseline LSER descriptor set was augmented with molar fractions of individual ionic species at pH 7.4, calculated within the same software environment. This combined descriptor workflow, computed LSER descriptors together with ionization-state correction, provides a standardized, reproducible, and scalable approach suitable for in silico virtual screening of structurally diverse compound libraries.^27,32^ Given the structural diversity of the analyzed compounds and the complex physicochemical nature of IAM retention, it was hypothesized that models capable of capturing both linear and nonlinear structure–retention relationships would outperform classical additive approaches. Therefore, a panel of regression algorithms spanning distinct computational paradigms was applied: MLR (linear, parametric baseline), SVR with linear and RBF kernels (regularized and kernel-based), kNN (instance-based), DT (rule-based, nonparametric), and the ensemble methods RF and LightGBM (bagging and gradient boosting, respectively). This selection reflects a deliberate continuum from simple to complex learning strategies, allowing systematic assessment of how different mathematical assumptions translate into predictive performance for LSER-based IAM retention modeling.

The predictive performance of all developed LSER–ML models is summarized in Table 1. To comprehensively assess the influence of molecular ionization on IAM retention, two descriptor configurations were compared: the baseline LSER model and the ionization-augmented LSER model incorporating the relative distribution of ionic species.

**Table 1:** Summary of Performance Metrics of the Obtained Model

model	*R* ^2^_train_	RMSE_train_	*Q* ^2^_cv_	*Q* ^2^_cv_ (SD)	RMSE_cv_	*R* ^2^_test_	RMSE_test_
LSER							
MLR	0.704	8.873	0.698	0.032	8.921	0.673	6.850
SVR-linear	0.700	8.936	0.695	0.026	8.972	0.668	6.902
kNN	0.811	7.090	0.683	0.061	9.117	0.792	5.457
SVR-RBF	0.821	6.907	0.756	0.063	7.967	0.780	5.613
DT	0.874	5.796	0.622	0.074	9.919	0.740	6.101
RF	0.947	3.766	0.741	0.056	8.217	0.834	4.874
LightGBM	0.881	5.641	0.735	0.046	8.356	0.774	5.697
LSER + Ionization Descriptors							
MLR	0.762	7.943	0.752	0.036	8.044	0.668	6.931
SVR-linear	0.756	8.051	0.742	0.032	8.218	0.681	6.788
kNN	0.816	6.993	0.697	0.035	8.906	0.829	4.967
SVR-RBF	0.884	5.546	0.811	0.048	6.989	0.853	4.609
DT	0.891	5.381	0.634	0.044	9.801	0.781	5.631
RF	0.971	2.778	0.778	0.028	7.619	0.851	4.648
LightGBM	0.928	4.383	0.777	0.048	7.605	0.809	5.256

Comparison of all approaches clearly showed that incorporating ionization-related information consistently improved model performance across both cross-validation and external validation stages, increasing the *R* ^2^_test_ and *Q* ^2^_cv_ coefficients by approximately 0.06–0.08 relative to the baseline LSER model. Li et al.^44^ showed that adding ionization-related properties enhances the description of solute-phospholipid interactions in IAM chromatography. The improvement in *Q* ^2^_CV_ observed upon incorporating ionization descriptors was assessed for statistical significance using a one-sided paired permutation test (10 000 permutations) with Benjamini–Hochberg correction for multiple comparisons. The addition of ionization descriptors yielded a statistically significant improvement across five of seven algorithms (MLR, SVR-linear, SVR-RBF, RF, LightGBM; p_BH_ < 0.05), with Δ*Q* ^2^_CV_ ranging from 0.032 to 0.063. The two methods without ensemble averaging (DT, kNN) did not reach significance (p_BH_ > 0.05), consistent with their generally lower predictive performance. Full results for the permutation test are provided in Table S3. Therefore, subsequent analyses focused on the ionization-augmented descriptor set as the most representative and robust configuration.

Nevertheless, it should be noted that models trained solely on LSER parameters still exhibited acceptable predictive ability, with the best one achieving a *Q* ^2^_test_ of 0.756. These findings are consistent with observations previously reported by Valkó et al.,^38^ who noted that only strongly ionized acids tend to behave as outliers in classical linear LSER models.

To provide mechanistic transparency and facilitate direct comparison with previously reported linear solvation equations for IAM chromatography, the system constants from the MLR model trained on the ionization-augmented descriptor set are presented below. This equation extends the classical LSER framework (eq 1) by incorporating four additional terms representing the molar fractions of ionic species at pH 7.4 (*n* = 794, *R* ^2^ = 0.762, RMSE = 7.943):

7 $${\mathrm{CHI}}_{\mathrm{IAM}}=\left[38.76\left(\pm 0.28\right)\right]+\left[9.74\left(\pm 0.86\right)\right]E-\left[5.22\left(\pm 0.91\right)\right]S-\left[0.61\left(\pm 0.37\right)\right]A,-[13.82\left(\pm 0.70\right)]B+\left[17.11\left(\pm 0.57\right)\right]V-\left[222.94\left(\pm 90.36\right)\right]f_{\mathrm{neutral}}-\left[182.79\left(\pm 75.04\right)\right]f_{\mathrm{cation}}-\left[199.50\left(\pm 79.52\right)\right]f_{anion}-\left[71.07\left(\pm 28.33\right)\right]f_{\mathrm{zwitterion}}$$

for Abraham descriptors *E* (excess molar refraction), *S* (dipolarity/polarizability), *A* (hydrogen-bond acidity), *B* (hydrogen-bond basicity), and *V* (McGowan characteristic volume), respectively, and the remaining terms are system constants associated with the molar fractions of neutral, cationic, anionic, and zwitterionic species at pH 7.4.

Analysis of the obtained results revealed clear and systematic patterns in the model learning behavior. In general, the linear models (MLR and SVR-linear) demonstrated noticeably lower predictive performance (*Q* ^2^_cv_ = 0.752 and 0.742, respectively), confirming the limited ability of purely additive relationships to describe IAM retention. In contrast, DT and more advanced algorithms such as RF and LightGBM exhibited a strong tendency toward overfitting. These models achieved a near-perfect fit to the training data (*R* ^2^_train_ = 0.891, 0.971, and 0.928, respectively) but showed poorer generalization, which was clearly visible in the cross-validation results (*Q* ^2^_cv_ = 0.634, 0.778, and 0.777, respectively). Notably, this overfitting tendency persisted for all tree-based models despite extensive hyperparameter tuning across the full grids reported in section 2.4.3, including restrictive settings for tree depth and minimum leaf size, suggesting that for a data set of this size and dimensionality, the capacity of tree-based ensembles exceeds what the available training data can effectively constrain, even under aggressive regularization. Similarly, the kNN algorithm, although demonstrating a balanced performance between training and external validation (*R* ^2^_train_ = 0.816 and *Q* ^2^_cv_ = 0.829), showed substantially lower cross-validation statistics (*Q* ^2^_cv_ = 0.697), indicating that its predictive reliability decreases when applied outside the most densely populated regions of the descriptor space.

Among all tested algorithms, the SVR-RBF model with the radial basis function kernel provided the most balanced performance across all validation levels, achieving *Q* ^2^_cv_ = 0.811 in cross-validation, *R* ^2^_train_ = 0.884 for the training set, and *R* ^2^_test_ = 0.851 for the external test set. To quantify this balance, three complementary selection criteria were applied. First, *Q* ^2^_cv_ was prioritized as the primary indicator of generalizability, since it averages predictive performance over five independent validation folds and is therefore less sensitive to the composition of a single hold-out set than *R* ^2^ test. SVR-RBF achieved the highest *Q* ^2^_cv_ (0.811), exceeding RF (0.778) and LightGBM (0.777). Second, the discrepancy between *R* ^2^ train and Q^2^_cv_ was used as a diagnostic for overfitting: this gap amounted to only 0.073 for SVR-RBF (0.884–0.811), compared to 0.193 for RF (0.971–0.778), indicating substantially tighter agreement between calibration and cross-validated performance. Third, *R* ^2^_test_ values for SVR-RBF (0.853) and RF (0.851) were essentially identical, confirming that the apparent superiority of RF in training accuracy does not translate into better external predictivity. The agreement between experimental and predicted CHI_IAM_ values is clearly illustrated in Figure 2A, where the data points are closely distributed along the identity line, indicating both good calibration and reliable external predictivity. The obtained results clearly indicate that the regularized kernel-based approach effectively captured the nonlinear relationships between LSER descriptors while maintaining model stability and avoiding overfitting. Calculated prediction errors were within the expected analytical uncertainty of the experimental method. According to Valkó et al.’s protocol,^38^ the acceptable analytical deviation for CHI_IAM_ is approximately ±5 units. The RMSE values obtained for the SVR-RBF model, 4.609 for the test set, 6.989 for cross-validation, and 5.546 for the training set, are very close to the experimental tolerance range, indicating that the model accuracy approaches the intrinsic reproducibility limit of the IAM method.

**Figure 2:**
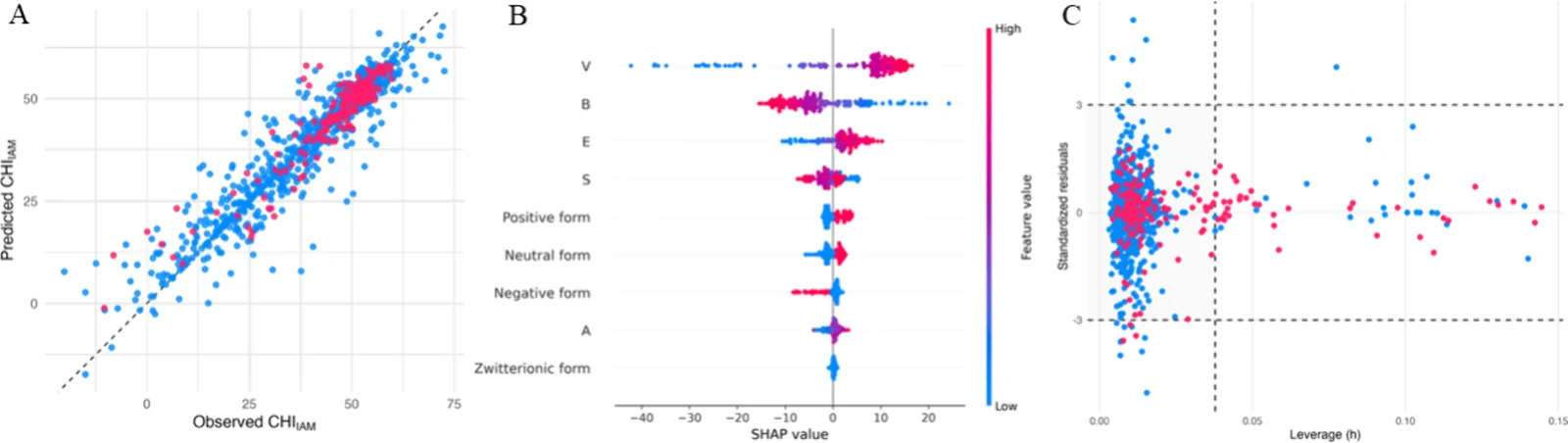
Performance and interpretability of the SVR-RBF model. (A) Plot of observed vs predicted CHI_IAM_ with the 1:1 reference line: training compounds, blue dots; test compounds, red dots. (B) SHAP summary showing feature contributions to CHI_IAM_. Color encodes feature value (low to high). (C) Williams plot (standardized residuals vs leverage) defining the applicability domain. Horizontal lines indicate ±3 residuals, and the vertical line marks the leverage threshold h* = 0.0378.

Based on the results obtained, the SVR-RBF model was selected for validation according to the principles recommended by the OECD. The OECD framework considers not only the statistical measures of internal and external performance but also the assessment of model interpretability and applicability domain. In this context, SHAP was employed to interpret the model, decomposing each prediction into additive contributions of individual molecular descriptors.^35^ An exact algorithm for computing Shapley values for SVM models with the RBF kernel has been reported;^45^ however, that formalism is explicitly derived for binary fingerprint features, for which each differing feature increments the squared Euclidean distance by a fixed unit, and its authors note that nonbinary (integer-based) representations would require adjustments that are not developed therein. In addition, the SVERAD scheme expresses feature contributions for SVM classification through class-label-scaled support-vector terms and log-odds, whereas the present task is regression. As the model operates on continuous physicochemical descriptors in a regression setting, SVERAD is not directly applicable, and SHAP values were therefore computed using the model-agnostic KernelExplainer (shap.KernelExplainer), which estimates Shapley values via weighted least-squares regression over feature coalitions.^46^ Because the descriptor set comprises only nine features, the full space of 2^9^ = 512 feature coalitions was enumerated, so the reported values represent the exact Shapley values of the model output for the chosen background rather than a sampled approximation. The resulting global descriptor rankings were independently corroborated by permutation importance (Figure S1), supporting the robustness of the mechanistic interpretation. The SHAP projection is presented in Figure 2B, illustrating how the relative importance and direction of influence of each descriptor contribute to the overall prediction patterns.

Consistent with the classical Abraham solvation framework, the McGowan characteristic volume (*V*) and hydrogen-bond basicity (*B*) were identified as the most influential variables, showing the largest SHAP magnitudes. This observation aligns with the coefficients reported in Valkó et al.’s linear solvation equation for CHI_IAM_, where the volumetric and hydrogen-bond basicity terms were shown to play dominant roles. This ranking was independently confirmed by permutation importance analysis (Figure S1), with all top-ranked features showing statistically significant importance. These results confirm that IAM retention is strongly governed by hydrophobic volume effects and modulated by hydrogen-bond acceptor strength. Positive SHAP values for large *V* descriptors indicate that bulkier and more hydrophobic molecules contribute positively to IAM retention, reflecting enhanced dispersion and van der Waals interactions with the phosphatidylcholine moieties. In contrast, negative SHAP values associated with *B* suggest that strong hydrogen-bond acceptors experience reduced retention due to less favorable interactions with the zwitterionic headgroups of the IAM phase, which is consistent with the negative sign of the hydrogen-bond basicity term in Valkó et al.’s equation.^38^ Moderate contributions from the *E* (excess molar refraction) and *S* (dipolarity/polarizability) descriptors indicate that polarizability and dipole interactions also influence retention, but to a lesser extent than hydrophobic and hydrogen-bonding effects. The additional descriptors encoding ionization state (neutral, positive, negative, and zwitterionic forms at pH 7.4) provide a mechanistic complement. Molecules existing predominantly in the neutral or positively charged forms exhibited positive SHAP values, confirming a stronger affinity for the phospholipid surface. Indeed, analysis of the present data set confirmed that compounds existing predominantly as cations at pH 7.4 are significantly more retained than neutral species (mean CHI_IAM_ = 46.84 vs 40.13, *p* < 0.001), while anions show reduced retention (mean CHI_IAM_ = 35.29, *p* < 0.001). This pattern is consistent with the pH-piston hypothesis,^47^ which predicts that positively charged species can interact favorably with the negatively charged phosphate groups located in the inner region of the phospholipid layer, thereby compensating or even exceeding the partitioning loss expected from ionization. Similar observations were reported by Grumetto et al.,^48,49^ who demonstrated that basic drugs are often more strongly retained on IAM phases than neutral lipophilic compounds. In contrast, strongly deprotonated acids exhibit reduced retention due to electrostatic repulsion, consistent with the observations of Valkó et al.^38^

Overall, the SHAP analysis demonstrates that the ML-extended LSER model reproduces the mechanistic trends captured in the original linear equation and simultaneously resolves subtle nonlinear relationships among molecular polarity, charge distribution, and retention, particularly for partially ionized species under physiological conditions.

Finally, the model’s applicability domain was evaluated using the Williams plot (Figure 2C), which relates standardized residuals to leverage values to identify potential outliers and influential observations. In the present case, only a small fraction of compounds fell outside the ±3σ residual threshold, corresponding to 2.14% of the training set and 1.51% of the validation set, indicating high overall predictive stability. Additionally, 6.14% of molecules exhibited leverage values exceeding the critical limit equal to 0.0378 but remained within the acceptable error range, showing low standardized residuals and accurate predictions. Although these compounds occupy less populated regions of descriptor space, they are still correctly predicted by the model. This behavior indicates a certain degree of extrapolation, which is particularly valuable for predictive modeling applications where generalization beyond the training chemical space is essential. Collectively, these results indicate that the developed LSER-ML framework is well-suited for integration into early drug discovery workflows, where CHI_IAM_ predictions for novel, uncharacterized compounds can support the prioritization of candidates with favorable membrane partitioning profiles, guide property-based molecular design, and complement experimental PAMPA or volume-of-distribution screening campaigns.

## 4 Conclusions

This study demonstrates that the classical LSER framework can be successfully combined with ML algorithms to describe and predict solute retention in biomimetic chromatographic systems. The inclusion of ionization-related parameters significantly improved the model’s predictive accuracy, confirming the importance of electrostatic interactions in IAM retention. Among all tested algorithms, the SVR-RBF model provided the best balance between calibration and generalization, achieving root-mean-square errors comparable to the experimental uncertainty. SHAP-based interpretation revealed that the model captures the same physicochemical mechanisms as the traditional LSER equation, with retention primarily governed by hydrophobic volume and hydrogen-bonding basicity, while also accounting for nonlinear relationships related to molecular polarity and ionization. The developed LSER-ML approach, therefore, extends the applicability of the Abraham solvation model to a wider chemical domain, offering a predictive, interpretable, and reproducible tool for in silico screening of compounds with diverse acid–base properties.

## Supplementary Material



## Notes

Table S1 listing the SMILES notation of the studied compounds together with references to the papers describing their synthesis and their allocation to the training and test sets; Table S2 listing the data set used for model development, including the molecular descriptors and the experimental CHI_IAM_ values; Table S3 listing the results of the paired permutation test with Benjamini–Hochberg correction used to assess the statistical significance of the improvement in *Q* ^2^_cv_ upon the addition of ionization descriptors, reported per algorithm and broken down by ionic form for the SVR-RBF model; and Figure S1, showing the permutation importance plot in *R* ^2^ upon random permutation of each descriptor, evaluated on the test set (XLSX)
